# Dose-Dependent Induction of Murine Th1/Th2 Responses to Sheep Red Blood Cells Occurs in Two Steps: Antigen Presentation during Second Encounter Is Decisive

**DOI:** 10.1371/journal.pone.0067746

**Published:** 2013-06-28

**Authors:** Claudia Stamm, Julia Barthelmann, Natalia Kunz, Kai-Michael Toellner, Jürgen Westermann, Kathrin Kalies

**Affiliations:** 1 Center for Structural and Cell Biology in Medicine, Institute of Anatomy, University of Lübeck, Lübeck, Germany; 2 MRC Centre for Immune Regulation, Division of Immunity and Infection, University of Birmingham Medical School, Birmingham, United Kingdom; New York University, United States of America

## Abstract

The differentiation of CD4 T cells into Th1 and Th2 cells *in vivo* is difficult to analyze since it is influenced by many factors such as genetic background of the mice, nature of antigen, and adjuvant. In this study, we used a well-established model, which allows inducing Th1 or Th2 cells simply by low (LD, 10^5^) or high dose (HD, 10^9^) injection of sheep red blood cells (SRBC) into C57BL/6 mice. Signature cytokine mRNA expression was determined in specific splenic compartments after isolation by laser-microdissection. LD immunization with SRBC induced T cell proliferation in the splenic T cell zone but no Th1 differentiation. A second administration of SRBC into the skin rapidly generated Th1 cells. In contrast, HD immunization with SRBC induced both T cell proliferation and immediate Th2 differentiation. In addition, splenic marginal zone and B cell zone were activated indicating B cells as antigen presenting cells. Interestingly, disruption of the splenic architecture, in particular of the marginal zone, abolished Th2 differentiation and led to the generation of Th1 cells, confirming that antigen presentation by B cells directs Th2 polarization. Only in its absence Th1 cells develop. Therefore, B cells might be promising targets in order to therapeutically modulate the T cell response.

## Introduction

T helper lymphocytes differentiate into distinct subsets of different functional capabilities and the potential to produce cytokines (reviewed in [1]). A well-studied example of how cytokine producing CD4 T cell subsets regulate immune responses is the cell-mediated (Th1) versus humoral (Th2) immune response. Th1 cells are defined as cells preferentially secreting cytokines such as IFNγ supporting cell-mediated immune responses. In contrast, the Th2 subset produces cytokines such as IL-4 and IL-5, signals typically inducing B cell activation and Ig class switching. It is thought that the selective differentiation of either subset is established early during priming [2], [3]. The best-known factor influencing T helper cell differentiation is the binding affinity of the MHC class II/peptide-complex to the T cell receptor, with strong binding affinity inducing Th1 cells whereas lower binding affinities lead to the generation of Th2 cells. Even a change of a single amino acid in the T cell receptor can shift T cell differentiation from Th1 to Th2 [4], [5]. While effects of MHC-TCR affinities on T cell priming have been studied well *in vitro*, it is difficult to control for binding affinity *in vivo*. For example, in the well known *Leishmania major* infection model C57BL/6 mice develop a Th1 response and survive. In contrast, BALB/c mice develop a Th2 response and die. In this situation it is almost impossible to control the binding affinity of the T cell receptor to the MHC class II/peptide-complex, because the T cell receptor repertoire and the MHC haplotype differ between the two mouse strains. In addition, *Leishmania major* parasites continuously change the expression of own molecules during their differentiation and proliferation within host cells whereby the antigenic peptides, which are presented to T cells, change and may lead to the engagement of completely different T cell clones in the two mouse strains [6].

Further, in many experimental systems the addition of adjuvants complicates the situation, and it is well known that adjuvants modulate Th1 and Th2 polarization [7], [8] thereby potentially overriding the effects of binding affinity on T helper cell differentiation. A technical issue has also to be considered. Many T cell cytokines *in vivo* are produced in minute amounts. Therefore, T cell isolation and *in vitro* restimulation often have been used to infer which cytokines were produced at a certain time of T cell differentiation *in vivo*. In the present study we set out to analyze Th1 and Th2 differentiation *in vivo* avoiding these problems. Th1 and Th2 responses were induced in the same mouse strain (C57BL/6). Sheep red blood cells (SRBC), which are non-replicating antigens that directly reach the spleen and are cleared within hours [9], were injected intravenously to induce either a Th1 response (delayed type hypersensitivity (DTH) reaction) by low dose application (LD; 10^5^ SRBC) or a Th2 response (IgG production) by high dose application (HD; 10^9^ SRBC) [10], [11], [12]. To avoid unwanted effects from *in vitro* restimulation, the cytokine response was measured by combining two techniques that allow detection of very low-level cytokine expression. By using laser-microdissection we could focus on T cell differentiation within the T cell zone (TCZ). By using real-time RT-PCR the cytokine signal could be amplified exponentially [13].

We found that two encounters with antigen were necessary to induce Th1/Th2 polarization. Only after activation of antigen-specific B cells a Th2 response developed. This occurred after high dose priming with antigen and required an intact splenic architecture. In contrast, priming with a dose too low to activate B cells led to a Th1 response. Our results indicate that this dose-dependent induction of Th1/Th2 cells is not restricted to SRBC and may play a role also for other antigens.

## Materials and Methods

### Mice and Injections

Eight- to 12-week-old female wild type C57BL/6 mice or LTβR^−/−^ C57BL/6 mice were obtained from Charles River Breeding Laboratories, housed and bred in the central animal facility of the University of Luebeck. All experiments were done in accordance with the German Animal Protection Law and were approved by the Animal Research Ethics Board of the Ministry of Environment (Kiel, Germany, # V312-72241.1221-1 (53-5/07). SRBC (Labor Dr. Merk, Ochsenhausen, Germany) were washed and 200 µl 0.9% NaCl containing 10^5^ or 10^9^ SRBC were injected into the tail vein. The spleens were removed 1, 9, 24, 48, 72, 144, and 240 h after injection of SRBC, snap frozen, stored at −80°C and subjected to analysis (laser-microdissection, T and B cell proliferation and antigen arrival).

### DTH Response

To test for DTH 10^9^ SRBC in 40 µl 0.9% NaCl were injected subcutaneously into the right footpad 5 days after intravenous injection of SRBC. The increase in footpad swelling was analyzed between 1 and 4 days (caliper, 0–10 mm, Kroeplin GmbH, Schlüchtern). In some experiments, the footpads were removed 24 and 48 h after s.c. injection of SRBC, snap frozen and stored at −80°C.

### Assessment of Antigen Arrival

10^5^ or 10^9^ SRBC were labeled with CFSE according to the manufacturer’s protocol (Sigma-Aldrich, Germany) and were i.v. injected into the tail vein. The spleens were removed 1, 9 and 24 h after injection (n = 3) and immersion fixed in 4% paraformaldehyde in phosphate buffered saline. 10 µm thick cryosections were cut, stained with Biotin conjugated rat anti-mouse metallophilic macrophage monoclonal antibody (MOMA-1) (Acris Antibodies GmbH, Hiddenhausen) and visualized with Streptavidin Alexa Fluor® 555 (SAV-555; Invitrogen, Life Technologies, Germany).

### Histological Analysis

Cryo-sections (10 µm thick) were mounted on membrane-covered slides (Palm Membrane Slides, PEN membrane, 1 mm; Carl Zeiss AG, Germany) for laser-microdissection or on usual glass slides for histology and stored at −80°C. The staining with toluidine blue was performed as described [13], [14]. To visualize the T and B cell compartments of the spleen, the sections were stained immunohistochemically with either biotinylated mAbs TCRβ (αβ T cells) or mAbs B220 (B cells, both BD Biosciences). Proliferating cells were identified by staining for Ki-67 Ag and counted as described [14] (TEC-3; DakoCytomation, Denmark). To study the formation of GC the size of the Ki-67 stained area (red) and the B cell zone (blue) was assessed (Palm Microbeam Software, Carl Zeiss AG). Cryostat sections from footpad skin samples were stained by H&E according to standard protocols. Staining for T cells, granulocytes and macrophages was performed using mAbs against TCRβ (BD Biosciences), Gr-1 (Ly6G, RBC-8C5, ebioscience, Germany) and F4/80 (abcam, UK) as primary antibodies, and either alkaline phosphatase goat anti-rat IgG (Roth, Karlsruhe, Germany) or goat anti-hamster (abcam) as secondary Ab. Alkaline phosphate activity was visualized with Fast Blue (BB Salt, Sigma, Germany).

### Laser-microdissection and Real-time RT-PCR

Spleens of immunized mice were isolated 9, 24, 48 or 72 h after priming. To dissect the marginal zones, T cell zones and B cell zones of SRBC primed spleens a pulsed UV laser was used (Palm Microbeam; Zeiss microImaging GmbH, Germany). Isolated tissues were processed as described [13], [15]. The tissues (spleens or skin) were shock frozen immediately after isolation and not allowed to thaw during their preparation in order to prevent any degradation of RNA. All specimens were treated identically, which excludes any biases between the groups. Primer sequences, amplicon sizes and gene accession numbers are shown in table S1 (Table S1).

### SRBC-specific IgG Detection in Serum

Blood was collected 1, 2, 3, 6, and 10 days after priming, serum was prepared and IgG levels were assessed by ELISA according to the manufacturers protocol (Bethyl Laboratories Inc.). 96-well plates (MaxiSorb, Nunc A/S, Denmark) were coated overnight with 10^8^ SRBC in PBS, extensively washed before incubated with samples and HRP-conjugated antibody (goat anti-mouse IgG-Fc HRP conjugated, Bethyl Laboratories, USA). The ELISA was developed using TMB (Invitrogen, Germany), stopped and analyzed at 450 nm. Control sera were pooled from naive C57BL/6 mice. To obtain arbitrary IgG units the OD of sera from primed mice were divided by the OD of control sera.

### Adoptive Transfer of Lymphocytes

Spleens of HD and LD primed mice were removed five days after priming. Single-cell suspensions of spleens were prepared. Erythrocytes were removed by treatment with Tris-ammonium chloride for 10 min at 4°C. The cells were washed and resuspended in 0,9% NaCl. 4×10^7^ cells in 200 µl NaCl were injected i.v. into the tail vein of naïve C57BL/6 mice. Immediately after cell transfer, 10^9^ SRBC were injected into the right footpad and footpad swelling was assessed between 1 and 4 days.

### Statistical Analysis

To determine whether differences were statistically significant, the nonparametric Mann-Whitney-U-test for two independent samples was performed (Graph pad prism, version 5).

## Results

### DTH Reaction is Induced after LD but not after HD Application of SRBC

**Figure 1:**
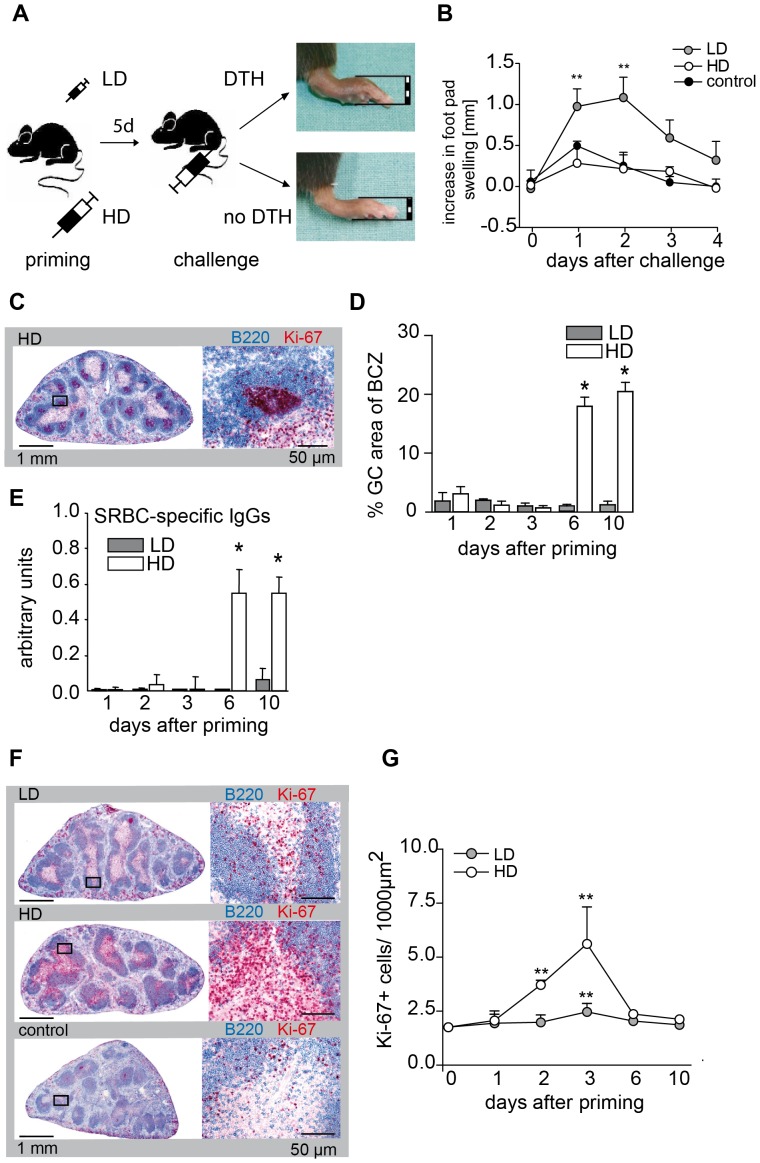
LD of SRBC induces DTH, HD the formation of GC. Mice were primed with a LD or a HD of SRBC intravenously. 5 days after priming one footpad was challenged with SRBC as described previously [10], [12]. The application scheme is shown in (**A**). Footpad thickness was measured between 1 and 4 days after challenge (*indicate significant differences in footpad thickness compared to control mice; n = 6–11; data were combined from three independent experiments with 2–5 mice) (**B**). Cryosections of spleens of LD and HD primed mice were stained immunohistochemically with antibodies against B cells (B220, blue). Proliferating cells were labeled with the proliferation marker Ki-67 (red) (**C** and **D**, **F** and **G**). Proliferating B cells within GC 10 d after HD priming are shown (**C**). The area of GC was measured and expressed as percentage of the area of the corresponding B cell follicles (*indicate significant differences between LD and HD primed mice at indicated time points; n = 3–6) (**D**). Serum from LD and HD primed mice was prepared and SRBC-specific IgGs were measured by ELISA (*indicate significant differences between LD and HD primed mice at indicated time points; n = 3–6) (**E**). Proliferating cells in spleens primed with a LD or HD and controls are shown (**F**). Proliferating cells were counted within the TCZ of LD and HD primed mice at indicated time points (*indicate significant differences between the number of proliferating T cells compared to unchallenged mice; n = 3–6) (**G**). All data are given as mean ± SD (*p<0.05; **p<0.01 Mann-Whitney-U-test). Abbreviations: BCZ, B cell zone; DTH, delayed type hypersensitivity reaction; GC, germinal center; HD, high dose (10^9^)*;* LD, low dose (10^5^); SRBC, sheep red blood cells.

The intravenous injection of heterologous red blood cells induces a T cell dependent immune response in the spleen. As described previously, mice that were primed by i.v. injection of 10^5^ SRBC (low dose, LD) develop a DTH when the footpad is challenged with the same antigen 5 days later (Fig. 1A and B) [10], [12]. When the dose of SRBC used for i.v. priming was raised to 10^9^ SRBC (high dose, HD) DTH disappeared completely in the footpad skin (Fig. 1B). Instead, HD priming induced the formation of germinal centers (GC) in the spleen (Fig. 1C and D) and the production of SRBC-specific IgG antibodies (Fig. 1E). Further analysis showed that both LD and HD priming with SRBC induced T cell proliferation in the TCZ of the spleen (Fig. 1F and G). T cell proliferation peaked 3 days after SRBC injection, but was 2–3 times higher when priming was done with a HD of SRBC (Fig. 1G). Thus, i.v. priming with both LD and HD SRBC induced a T cell proliferation in the spleen. However, only LD T cells were able to induce a DTH reaction in the footpad skin whereas only HD T cells provided help for GC formation.

### Only LD T cells Enter the Skin

**Figure 2:**
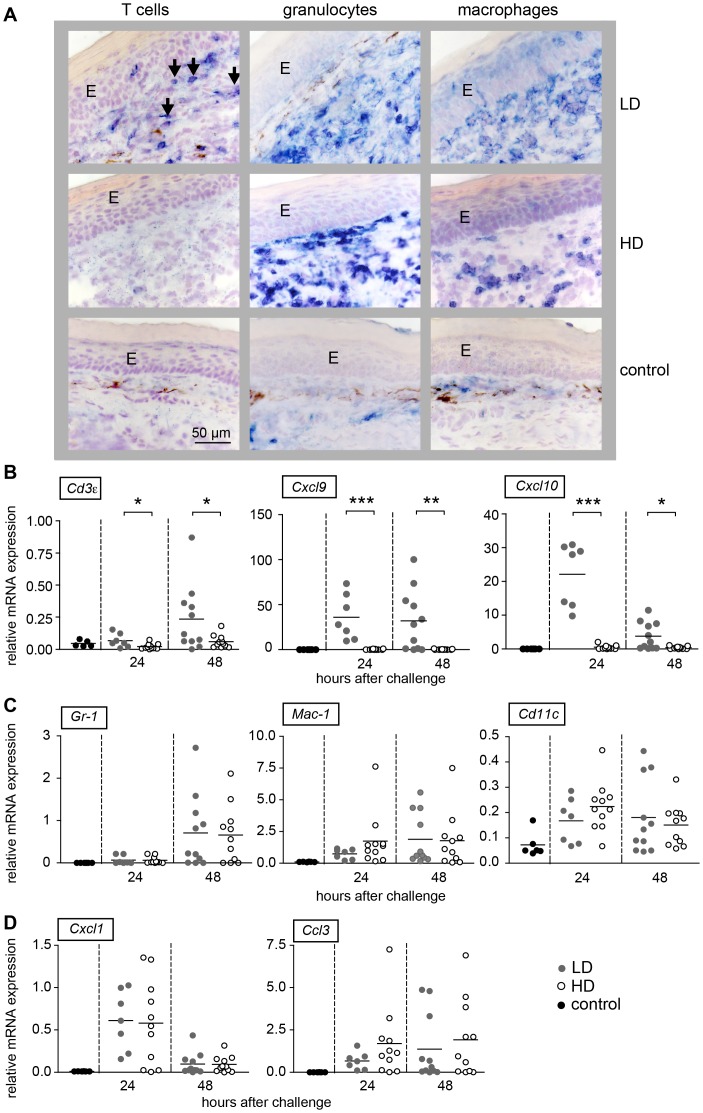
An increased recruitment of T cells was found only in footpads of LD primed mice. Mice were primed with a LD or a HD of SRBC intravenously. 5 days after priming one footpad was challenged with SRBC. Serial cryostat sections from footpad skin 48 h after challenge with SRBC and PBS treated control skin were stained for T cells (TCRβ, blue, left panel), granulocytes (GR-1, blue, middle panel) and macrophages (F4/80, blue, right panel) (bar: 50 µm) (**A**). It is interesting to note that the staining pattern for GR-1 and F4/80 looks different between LD and HD primed mice indicating distinct activation levels. For quantification, the mRNA expression of *CD3ε*, as T cell marker, *Cxcl9* and *Ccxl10* as T cell attracting chemokines (**B**), *Gr-1, Mac-1* and *Cd11c* as cell markers for innate cells (**C**) and the chemokines *Cxcl1* and *Ccl3* (**D**) was analyzed by real-time RT-PCR and normalized to the house keeping gene *Mln51* in the footpad skins 24 h and 48 h after challenge with SRBC. Each dot represents the expression level of one mouse. Corresponding means are depicted as black line. *indicate significant differences between the level of expression between HD and LD primed mice (*p<0.05, **p<0.01; ***p<0.001, Mann-Whitney-U-test, n = 5–11). Abbreviations: E, epidermis; HD, high dose (10^9^)*;* LD, low dose (10^5^).

To find out whether LD and HD T cells entered the footpad skin during DTH reaction we immunohistochemically stained footpad skin of LD and HD primed mice 48 h after injection of SRBC using TCRβ antibodies (Fig. 2A, left panel). Interestingly, TCRβ positive cells were found only in skin of LD primed mice. To quantify and confirm the presence of T cells we analyzed the expression of *Cd3ε*, *Cxcl9* and *Cxcl10* mRNA 24 and 48 h after injection of SRBC into the footpad (Fig. 2B *)*. CD3ε as part of the T cell receptor complex is a specific marker for T cells, whereas CXCL9 and CXCL10 are typical chemokines, which attract T cells to the skin. As expected, a significantly increased expression of *Cd3ε*, *Cxcl9* and *Cxcl10* mRNA was found only in LD primed mice (Fig. 2B). This shows that only LD T cells were able to enter the skin.

To assess whether innate effector cells like granulocytes and macrophages are recruited in the SRBC-bearing footpad skin of LD and HD primed mice we stained sections with anti-Gr-1 (marker for granulocytes) and anti-F4/80 (marker for macrophages) (Fig. 2A, middle and right panel). To quantify positive cells, we analyzed mRNA expression of *Gr-1* (expressed on granulocytes) and *Mac-1* (expressed on macrophages) and their corresponding chemokines *Cxcl1* (attracts granulocytes), *Ccl3* (recruits and activates macrophages) and *Cd11c* as marker for dendritic cells in footpad skin of LD and HD primed mice after challenge with SRBC. Interestingly, granulocytes and macrophages appeared in similar numbers in the skin of LD and HD primed mice (Fig. 2A, middle and right panel) (Fig. 2A). Correspondingly, the expression of *Gr-1* and *Mac-1* transcripts increased significantly after injection of SRBC into the footpad skin (Fig. 2C and D). However, no difference in mRNA expression was found between footpad skins of LD and HD primed mice (Fig. 2C and D), although only the former responded with a DTH reaction. This shows that in contrast to T cells, the recruitment of innate immune cells into the footpad skin proceeds similar in LD and HD primed mice.

**Figure 3:**
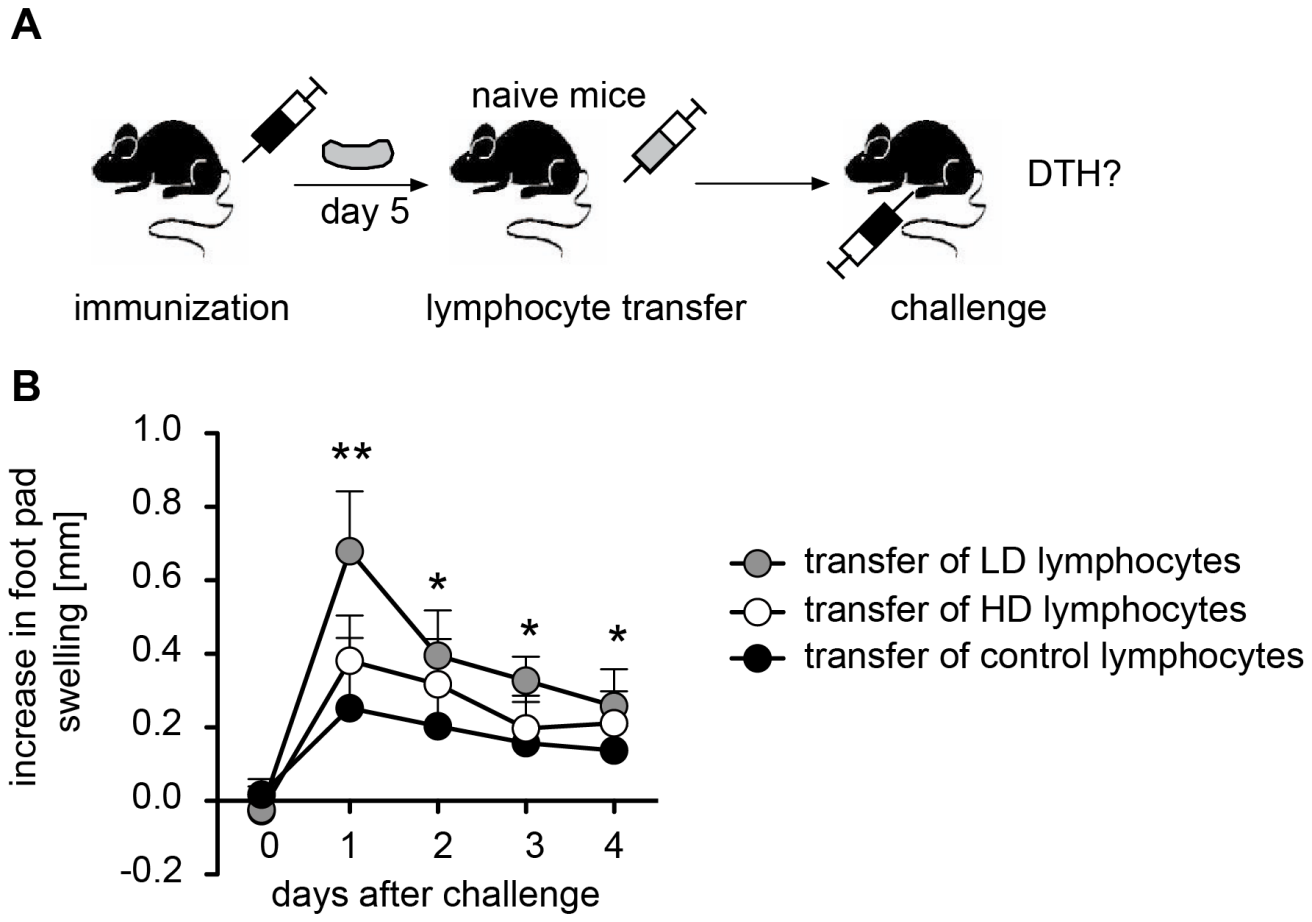
Only lymphocytes of LD primed mice are able to mount a DTH response. Mice were primed with a LD or a HD of SRBC intravenously. Spleens were removed 5 days after priming; cells were isolated and injected intravenously into naïve recipients. DTH was induced immediately by injection of SRBC into one footpad. The application scheme is shown in (**A**). Increase in footpad swelling was measured between 1 to 4 days after injection (**B**). Data are means ± SD. *indicate significant differences in footpad thickness compared to control mice at indicated time points (*p<0.05, **p<0.01; ***p<0.001, Mann-Whitney-U-test, n = 5–8). Abbreviations: DTH, delayed type hypersensitivity reaction; HD, high dose (10^9^)*;* LD, low dose (10^5^).

The absence of HD T cells in footpad skin despite much stronger proliferation in the spleen could be due to two principle mechanisms. They may be retained in the spleen. Alternatively, they are released into the blood but are unable to enter into the SRBC-bearing footpad skin. To differentiate between these possibilities we isolated T cells from the spleen of LD and HD primed mice and injected them intravenously into naïve recipients. The recipients were challenged by injection of SRBC into the footpad (Fig. 3A). As expected, LD T cells induced a DTH reaction (Fig. 3B). However, HD T cells were unable to induce a DTH reaction (Fig. 3B). This makes retention of HD T cells in the spleen unlikely and strongly indicates that HD T cells are not able to enter into the skin.

### LD T cells need a Second Contact with Antigen to become *Ifnγ* Expressing Th1 Cells

**Figure 4:**
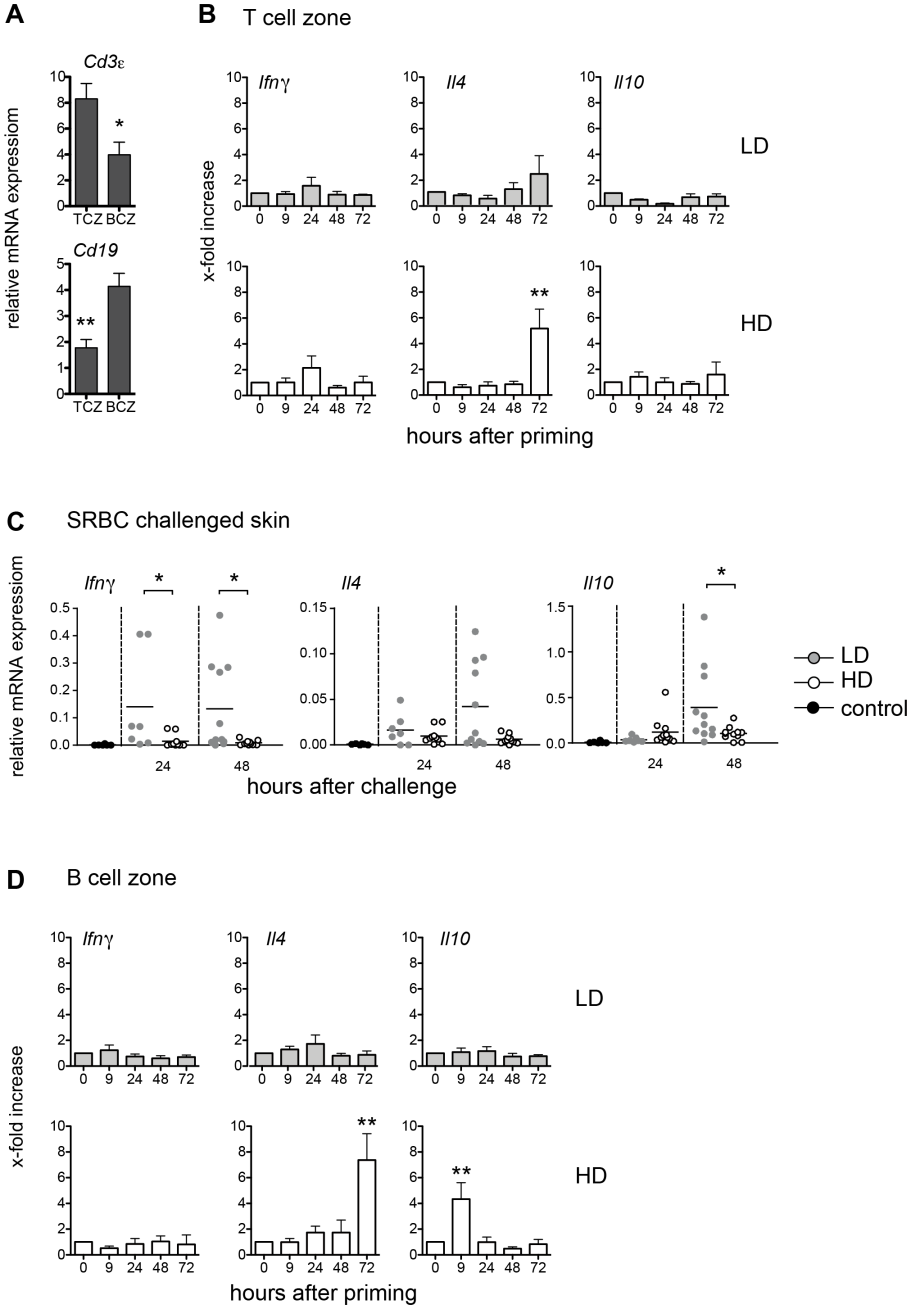
LD Th1 cells appear in the skin, HD Th2 cells in the spleen. Mice were primed with a LD or a HD of SRBC intravenously. The spleens were snap frozen, sections were prepared and the TCZ and BCZ were isolated by laser-microdissection. To confirm that the TCZ and BCZ are accurately identified and high-quality mRNAs are obtained, we analyzed mRNA expression of *Cd3ε* and *Cd19* after laser-microdissection (**A**). The mRNA expression of the Th1 cytokine *Ifnγ,* the Th2 cytokine *Il4* and the marker cytokine for regulatory T cells *Il10* was analyzed by real-time RT-PCR after isolation of the TCZ of the spleen. Data are means ± SEM. Data are normalized to *Mln51* mRNA expression levels. Significant differences in the expression of *Ifnγ, Il4* and *Il10* between primed mice compared to the controls are shown (n = 6, from two independent experiments with n = 3 mice) (**B**). 5 days after priming one footpad was challenged with SRBC. Mice were sacrificed 24 and 48 h after challenge and the expression of *Ifnγ, Il4* and *Il10* was analyzed in the footpad skins by real-time RT-PCR. Each dot represents the expression level of one mouse. Corresponding means are depicted as black line. Significant differences between LD and HD primed mice are shown (n = 5–11) (**C**). The mRNA expression of *Ifnγ, Il4* and *Il10* was analyzed by real-time RT-PCR after isolation of the BCZ of the spleen, and depicted as described in A (**D**) (*p<0.05, **p<0.01, Mann-Whitney-U-test). Abbreviations: HD, high dose (10^9^)*;* LD, low dose (10^5^).

To understand the molecular mechanisms by which LD and HD application of the same antigen induces two functionally distinct T cell populations we studied the expression of cytokines in the T cell zone (TCZ) and B cell zone (BCZ) of the spleen over time after i.v. priming with SRBC. To confirm the correct isolation of the TCZ and BCZ the mRNA expression of the T cell marker *Cd3ε* and the B cell marker *Cd19* was analyzed (Fig. 4A). The expression of *Ifnγ, Il4* and *Il10* mRNA were taken as marker cytokines for Th1 (DTH inducers), Th2 (GC inducers), and regulatory T cell populations, respectively. The TCZ was isolated by laser-microdissection and the mRNA expression was subsequently analyzed by real-time RT-PCR. This approach is not only highly sensitive, but also focuses directly on anatomical compartments. Surprisingly, despite the induction of T cell proliferation (Fig. 1G), no change in cytokine expression was detectable in the TCZ after priming with LD SRBC (Fig. 4B). In contrast, an increased expression of *Il4* was found in the TCZ 72 h after priming with a HD of SRBC. Obviously, LD T cells fail to develop into Th1 cells in the TCZ of the spleen whereas HD T cells directly differentiate into Th2 cells within the spleen (Fig. 4B). To test, whether during LD priming with SRBC the antigen is removed from the spleen too quickly to allow differentiation into cytokine producing effector T cells, we compared the expression of *Ifnγ, Il4* and *Il10* in the SRBC challenged footpad skin of LD and HD primed mice during DTH response. Interestingly, the expression of *Ifnγ* increased rapidly 24 h after injection of SRBC but only in the footpad of LD primed mice. In addition, the expression of *Il10* and of *Il4* increased 48 h after challenge; whereby the latter one did not reach statistical significance (Fig. 4C). Thus, LD T cells need a second contact with antigen in order to become cytokine expressing T effector cells.

### HD T cells need an Intact Splenic Architecture, Especially a Marginal Zone, to become *Il4* Expressing Th2 Cells

**Figure 5:**
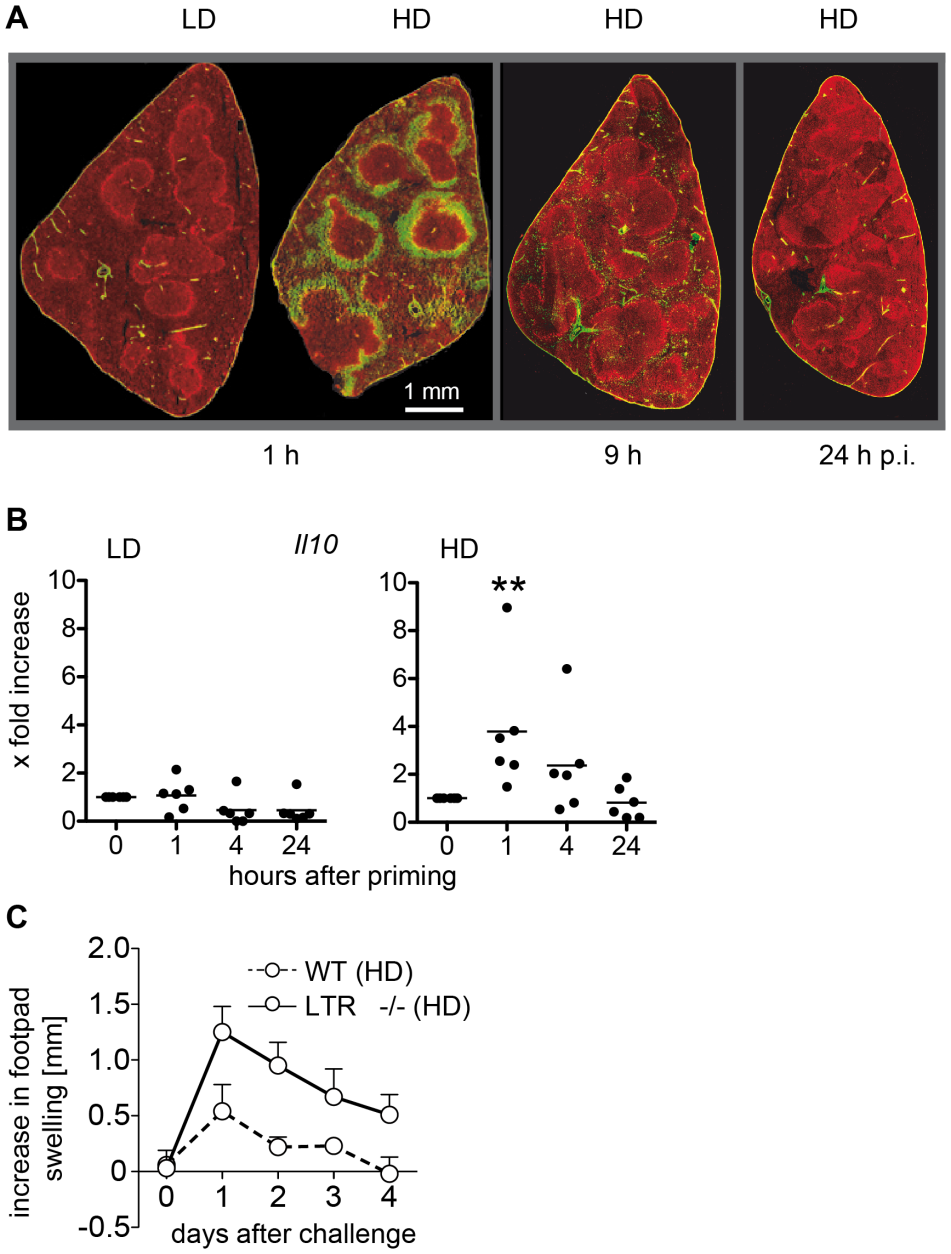
An intact MZ is required for the induction of Th2 cells. Mice were primed with a LD or HD of SRBC. SRBC were labeled with CFSE before injection (green) and spleens were removed 1 h, 9 h and 24 h after LD and HD priming. Histological sections were prepared and the MZ were visualized by staining with Alexa 555-labelled antibodies against Moma-1, which is specific for metallophilic macrophages (red) [37] (n = 3, one typical section is shown) (**A**). The MZ was isolated by laser-microdissection at indicated time points and mRNA expression of *Il10* as marker for activated MZ B cells was analyzed. Each circle represents one animal (n = 6) (**B**). Significant differences in the expression of *Il10* between primed mice compared to the controls are shown (**p<0.01, Mann-Whitney-U-test, n = 6). Mice were primed with a HD of SRBC. One footpad was challenged with SRBC 5 days after priming. The footpad thickness was measured between 1 and 4 days after challenge of wild type and LTβR−/− mice (**C**). Data are means ± SD. *indicate significant differences in footpad thickness compared to control mice (**p<0.01, Mann-Whitney-U-test, n = 6). Abbreviations: HD, high dose (10^9^)*;* LD, low dose (10^5^).

To delineate the molecular mechanisms that facilitate the development of HD T cells into *Il4* expressing cells, we analyzed the expression of cytokine mRNAs in the BCZ of the spleen. While no significant change in the expression of any cytokine was found after i.v. priming with a LD of SRBC, i.v. priming with a HD of SRBC induced strong expression of *Il4* in the BCZ after 72 h (Fig. 4D). Interestingly, we also found an early and transient increase in expression of *Il10* 9 h after HD priming (Fig. 4D). This early increase in *Il10* expression may indicate an activation of B cells by HD priming [16]. In turn, these activated B cells might present the antigen to HD T cells thereby facilitating their development into *Il4-*producing Th2 cells. Some of the *Il4-*expressing HD T cells subsequently enter the BCZ (increase in *Il4* 72 h after HD priming) (Fig. 4D). Since it is well known that antigens enter the spleen via the marginal zone (MZ) we asked whether during this phase signals are initiated that would induce Th1 or Th2 development in an antigen dose dependent fashion. SRBC were labeled with CFSE and i.v. injected. Only after HD application an accumulation of SRBC in the MZ was found (Fig. 5A) 1 h after priming, which disappeared within 24 h. The expression of *Il10* in the MZ did not change after LD priming. However, it increased significantly shortly (1 h) after HD priming reaching background levels again within 24 hours (Fig. 5B). Together, our data indicate that immediately after HD priming B cells in the MZ become activated (early increase of *Il10* in MZ; Fig. 5B). Subsequently, B cells in the BCZ get activated (early increase of *Il10* in BCZ; Fig. 4D) which are involved in inducing *Il4* expression by HD T cells (late increase of *Il4* in TCZ and BCZ; Fig. 4B and D). Thus, we hypothesized that the MZ is instrumental for the formation of HD T cells expressing *Il4*. Consequently, mice that lack the MZ would not develop *Il4*-expressing HD T cells. Instead, in those mice HD priming would induce the generation of T cells that keep their ability to enter the skin and to induce a DTH upon challenge with SRBC - very much like LD T cells. To test this, LTβR deficient mice [17] and wild type mice were primed with the HD regimen and five days later footpad skin was challenged with SRBC. As predicted, LTβR deficient mice, in which the MZ is missing and which were primed with a HD of SRBC, developed a DTH reaction whereas wild type mice did not (Fig. 5C).

## Discussion

Conflicting data exist regarding the role of the antigen dose on CD4 T cell differentiation [18]. Obviously, the type of antigen (soluble or particular), the route of administration and, most importantly, the method of data assessments (*in vitro* or *in vivo*) are crucial. In addition, even the outcome of *in vivo* experiments depends on many factors. Genetic background of the host [19], nature of the pathogen [20], antigen transport from tissue into the draining lymph node [21] and application of adjuvant [7], [8] affect Th1/Th2 development. In addition, these factors can influence each other. For example, adjuvant modulates the affinity with which the antigen is presented and thereby induces either Th1 (high affinity) or Th2 (low affinity) responses [7]. Furthermore, analysis of cytokine expression *in vitro* (as performed in most studies) can give almost opposite results depending on the mode of T cell stimulation [14]. With so many variables it is difficult to characterize the contribution of single factors to Th1/Th2 development *in vivo*. In the present study we combined a well-known model with an innovative technique to avoid such complications. By using SRBC as antigen the co-application of adjuvant was not necessary. SRBC induce a T cell dependent immune response, which is exclusively mediated by CD4 T cells [22]. In this system a clear polarization towards Th1 or Th2 responses can be induced in the same mouse strain (C57BL/6) just by varying the antigen dose (Fig. 1B and D) [10], [12]. SRBC are non-replicating particulate antigens, which are cleared within hours focusing the presence of the antigen to the priming phase of the immune response (Fig. 5A) [9]. Importantly, detecting T cell activation using the combination of laser-microdissection and RT-PCR reflects actual cytokine production and T cell effector function *in vivo*, without the need of restimulation *in vitro* reducing the occurrence of possible artifacts. Further, by using laser-microdissection, the analysis can be focused on cytokine production in either the TCZ or BCZ [13], [15].

### Th1/Th2 Differentiation Needs Two Antigen Encounters

The present study confirms that LD priming with SRBC induces a cellular (Th1) response (Fig. 1B) [10], [12], [23]. However, expression of *Ifnγ*, hallmark of a Th1 response, is not seen in the splenic TCZ (Fig. 4B). Instead, T cells and *Ifnγ* expression are found in the footpad skin 24 h and 48 h after challenge with SRBC (Fig. 4C). This rapid increase in *Ifnγ* expression indicate the arrival of antigen-specific pioneer T cells, which are typical for DTH responses and which are required to condition the tissue for further recruitment of specific and unspecific effector cells [24]. The further recruitment and activation of other effector cells, which is restricted to the skin of LD primed mice, is reflected by the increased expression of *Il4* and *Il10* at later time points (48 h). The fact the spleen is needed to mount a DTH after i.v. LD priming with SRBC [12] indicates that T cell proliferation induced by LD priming in the splenic TCZ (Fig. 1F and G) is necessary but not sufficient to cause T cell differentiation. The present study shows that a second contact with the antigen is needed to accomplish T cell differentiation, which after LD application takes place in a non-lymphoid organ, the skin (Fig. 4C). It is known that T cells need an additional contact with antigen in order to proceed from a cell producing the cytokine only at the mRNA level to a cell secreting the actual protein [25]. Here, we demonstrate that after SRBC application activated T cells need to see the antigen twice to be able to express signature cytokines (*Ifnγ* in the skin, *Il4* in the BCZ) even at mRNA level. Although it is possible that already after first antigen encounter T cell polarization has occurred in the TCZ with cytokine mRNA levels being below our detection limit, this seems unlikely because *Ifnγ* transcription is detected rapidly in the SRBC bearing skin even though few SRBC specific T cells are present at this site. Thus, our data indicate that the second antigen encounter induces a qualitative change in cytokine expression, in any case, however, a significant change in quantitative terms. Similar results were found in the *Leishmania major* infection model [14]. In this model BALB/c mice respond by producing Th2 cells whereas C57BL/6 mice develop a Th1 response. Interestingly, in BALB/c mice much more *Leishmania major* parasites were found in the BCZ of infected lymph nodes compared to those of C57BL/6 mice (data not shown). This suggests that early after infection the high parasite load in the BCZ of BALB/c mice may trigger the activation of *Leishmania major* specific B cells, which in turn induces Th2 cells, very similar to the scenario obtained in mice primed with a HD of SRBC.

### Antigen Presentation by B cells is Essential for Th2 Development

For HD priming with SRBC the situation is different. Here after 9 h, we already see an increase in *Il10* expression in the BCZ indicating an activation of B cells (Fig. 4D), which is down regulated within 24 h after priming. This temporary nature of the increase in *Il10* transcription reflects the removal of SRBC from the spleen (Fig. 5A). However, interestingly, an increased expression of *Il4* appears in both the BCZ and the TCZ 72 h after priming. This suggests that initial T cell activation by dendritic cells without an immediate secondary stimulus induces Th1 polarization, whereas subsequent antigen presentation from B cells induces Th2 differentiation. This conclusion is supported by several other observations: inhibition of B cells with cyclophosphamide during HD priming results in a Th1 response [23], [26]. On the other hand, a Th2 response can be induced by LD priming, when activated, SRBC-specific B cells are adoptively transferred before the skin is challenged with SRBC [26]. Furthermore, the role of B cells in inducing a Th2 response is not restricted to the SRBC model. The Th2 response developing in *Leishmania major* infection model of susceptible BALB/c mice is strongly impaired when B cell deficient BALB/c mice are infected [27], [28].

### Th2 cells Lose the Ability to Enter the Skin

Our experiments show that the lack of DTH after HD priming is neither due to suppression nor to clonal exhaustion, as T cells do proliferate in the TCZ and induce formation of GC (Fig. 1D and G). Our data show that HD T cells do not enter the skin (Fig. 2A and B). When LD T cells and HD T cells are adoptively transferred, only LD T cells but not HD T cells induce a DTH (Fig. 3). This DTH response is shortened (Fig. 3) compared to the response in unmanipulated mice (Fig. 1B) due to the limited number of LD T cells in the blood. However, HD T cells directly injected into the footpad skin are able to mount a DTH reaction [29]. The lack of skin homing of HD T cells is probably due to the lack of expression of the chemokine receptor CXCR3, which is essential for entry of Th1 cells into the skin in response CXCL9 and CXCL10 [30], [31].

### An Intact Splenic Architecture is Required for Th2 Induction

After HD priming SRBC quickly accumulate in large numbers in the MZ and significantly increase *Il10* expression within one hour of injection (Fig. 5A and B). Since earlier studies showed that binding of SRBC in the MZ of rat spleens was not associated with macrophages we conclude that this increase in *Il10* expression indicates an activation of IL10 producing CD1hi CD5+ marginal zone B cells (B10 cells) [16], [32]. Once activated, marginal zone B cells can migrate into the BCZ, deposit antigen there and induce the activation of SRBC-specific B cells allowing them to present the antigen to SRBC-specific T cells thereby completing Th2 differentiation [33], [34], [35]. Correspondingly, this Th2 differentiation is suppressed in LTβR^−/−^ mice, which miss MZ B cells and follicular DC [17]. However, histological analysis revealed that the size of the BCZ is unchanged, that of the mixed TCZ/BCZ is enlarged and that of the TCZ is reduced [17]. Our results demonstrate that T cells have access to SRBC and respond to it (Th1, Fig. 5C). In contrast, the Th2 response is impaired even though B cells are located in close proximity to the T cells.

In conclusion, this shows that the development of Th2 responses depends on lymphoid compartments that concentrate and direct antigen towards secondary B cell mediated antigen presentation, whereas without this, secondary antigen encounters in the periphery lead to fast onset of Th1 effector cytokine expression. Thus, it might be a promising approach to influence T cell responses via their interaction with B cells [36].

## Supporting Information

Table S1 **Primer sequences, amplicon sizes, and gene accession numbers of the analyzed genes^a^.** *a* Information obtained from the National Resource for Molecular Biology Information (www.ncbi.nlm.nih.gov). *b* for, forward; rev, reverse.
(DOCX)
