# Supplementary material for: Dose-Dependent Induction of Murine Th1/Th2 Responses to Sheep Red Blood Cells Occurs in Two Steps: Antigen Presentation during Second Encounter Is Decisive
Source: PLoS One. 2013 Jun 28;8(6):e67746. doi: 10.1371/journal.pone.0067746 (PMC3695941; doi:10.1371/journal.pone.0067746)
Supplement: Table S1 — Primer sequences, amplicon sizes, and gene accession numbers of the analyzed genesa. a Information obtained from the National Resource for Molecular Biology Information (www.ncbi.nlm.nih.gov). b for, forward; rev, reverse. (DOCX) [file pone.0067746.s001.docx]

**Table S1.**

Primer sequences, amplicon sizes, and gene accession numbers of the analyzed genes^a^

| Oligo Name | 5' Nucleotide Sequence | Size (bp) | Accession Number |
| --- | --- | --- | --- |
| MLN51 for*^b^* | ccaagccagccttcattcttg | 134 | NM_138660.2 |
| MLN51 probe | cacgggaacttcgaggtgtgcctaac |  |  |
| MLN51 rev | taacgcttagctcgaccactctg |  |  |
| CCL3 for | cactctgcaaccaagtcttc | 138 | NM_011337 |
| CCL3 Probe | ccatatggagctgacaccccgactgc |  |  |
| CCL3 rev | cacctggctgggagcaaag |  |  |
| CD3ε for | ataggaaggccaaggccaag | 145 | NM_007648.3 |
| CD3ε probe | ccagactatgagcccatccgcaaagg |  |  |
| CD3ε rev | tcaggccagaatacaggtc |  |  |
| CD19 for | gaaaatgcagatgaggagctgg | 145 | NM_009844.1 |
| CD19 probe | caaccagttggcaggatgatggacttctt |  |  |
| Cd19 rev | gctgcatagaggatccctctc |  |  |
| CD11c for | ccactgtctgccttcatattc | 95 | NM_021334.2 |
| CD11c rev | gacggccatggtctagag |  |  |
| CXCL1 for | cagaccatggctgggattc | 121 | NM_008176.2 |
| CXCL1 Probe | cctcgcgaccattcttgagtgtggcattgac |  |  |
| CXCL1 rev | gaaccaagggagcttcag |  |  |
| CXCL9 for | cttatcactagggttcctcgaactccacac | 72 | NM_008599 |
| CXCL9 probe | ttgggcatcatcttcctggag |  |  |
| CXCL9 rev | gcaggagcatcgtgcattc |  |  |
| CXCL10 for | cggattcagacatctctgctc | 106 | NM_021274.2 |
| CXCL10 probe | catcgtggcaatgatctcaacacgtgg |  |  |
| CXCL10 rev | gagggccatagggaagcttg |  |  |
| GR1 for | gcgttgctctggagatagaag | 127 | NM_010742 |
| GR1 rev | cttcacgttgacagcattacc |  |  |
| IFNγ for | gcaaggcgaaaaaggatgc | 98 | NM_008337.2 |
| IFNγ probe | tgccaagtttgaggtcaacaacccacag |  |  |
| IFNγ rev | gaccactcggatgagctcattg |  |  |
| IL4 for | gagactctttcgggcttttcg | 96 | NM_021283.1 |
| IL4 probe | cctggattcatcgataagctgcaccatg |  |  |
| IL4 rev | aggctttccaggaagtctttcag |  |  |
| IL10 for | tccctgggtgagaagctgaag | 96 | NM_010548.1 |
| IL10 probe | ctgaggcgctgtcatcgatttctccc |  |  |
| IL10 rev | cacctgctccactgccttg |  |  |

*a* Information obtained from the National Resource for Molecular Biology Information ([www.ncbi.nlm.nih.gov](http://www.ncbi.nlm.nih.gov)). *b* for, forward; rev, reverse.
